# Biochemical Pseudoprogression in Pancreatic Cancer During Chemotherapy: A Case Report

**DOI:** 10.7759/cureus.97465

**Published:** 2025-11-21

**Authors:** Abdolhakim Mohamed, Kevin T Dao, Stanley Kim

**Affiliations:** 1 Medicine, Ross University School of Medicine, Miramar, USA; 2 Internal Medicine, Kern Medical, Bakersfield, USA; 3 Hematology and Oncology, MacNeal Cancer Center, Loyola University Chicago Stritch School of Medicine, Berwyn, USA

**Keywords:** biochemical pseudoprogression, ca19-9, elevated ca19-9, pancreatic cancer, pseudoprogression

## Abstract

Pancreatic ductal adenocarcinoma (PDAC) is a highly lethal malignancy, most often diagnosed at an advanced stage, with limited treatment options and poor outcomes. CA 19-9 is the most commonly used biomarker to support the diagnosis and for treatment monitoring of PDAC, but rising levels during treatment usually suggest disease progression. Pseudoprogression, a transient increase in tumor burden or biomarker levels followed by subsequent improvement, has been described in glioblastoma and in cancers treated with immune checkpoint inhibitors (ICIs), but to our knowledge, it has not previously been reported in pancreatic cancer treated with cytotoxic chemotherapy.

We describe a 52-year-old man with metastatic PDAC who was treated with FOLFOX due to underlying cardiac comorbidities. Despite rising CA 19-9 levels from 2,344 U/mL at baseline to more than 90,000 U/mL after two months of therapy, the patient experienced clinical improvement, with resolution of abdominal pain, regained appetite, weight recovery, and enhanced performance status. Continued treatment resulted in a subsequent decline and plateau of CA 19-9 levels, consistent with biochemical pseudoprogression. This case represents a unique and previously undocumented phenomenon of pseudoprogression in PDAC under chemotherapy, underscoring the importance of integrating clinical status with biomarker interpretation to avoid premature discontinuation of effective treatment.

## Introduction

Pancreatic ductal adenocarcinoma (PDAC) accounts for the majority (90%) of pancreatic neoplasms. Its incidence is rising at a rate of 0.5% to 1.0% per year, and pancreatic cancer is projected to become the second-leading cause of cancer death in the U.S. by 2030. Most patients with pancreatic cancer are diagnosed at an advanced stage that is not amenable to curative surgery [1]. For patients with metastatic disease, systemic chemotherapy with modified FOLFIRINOX (fluorouracil, oxaliplatin, leucovorin, and irinotecan) or a combination of paclitaxel and gemcitabine is the first-line therapy. Milder but still effective FOLFOX is often used as a second-line therapy when patients fail paclitaxel and gemcitabine [2]. CA 19-9 is the most commonly used biomarker to support the diagnosis, and CA 19-9 levels during and after chemotherapy can predict a patient’s response to treatment [3]. However, as CA 19-9 is a sialylated Lewis blood group antigen, approximately 6% of the White population and 22% of the Black population in the U.S., who are Lewis antigen-negative, do not produce CA 19-9, even if they have advanced pancreatic cancer [3].

Pseudoprogression is defined as an objective response following initial progression with the same treatment [4], which is clinically manifested as a temporary increase in specific biochemical markers or tumor size during therapy, mimicking true disease progression without indicating a worsening condition. Pseudoprogression has been observed in various cancers treated with immune checkpoint inhibitor (ICI) immunotherapy, mostly in melanoma [4-6]. For instance, prostate-specific antigen (PSA) levels may temporarily rise in prostate cancer patients after starting immunotherapy [5]. However, pseudoprogression has not been seen in metastatic colorectal or pancreatic cancer treated with immunotherapy in clinical studies, nor has it been reported in pancreatic cancer treated with cytotoxic chemotherapy [6]. We present a unique case of biochemical pseudoprogression of metastatic PDAC during active chemotherapy, mimicking actual disease progression.

## Case presentation

**Figure 1:**
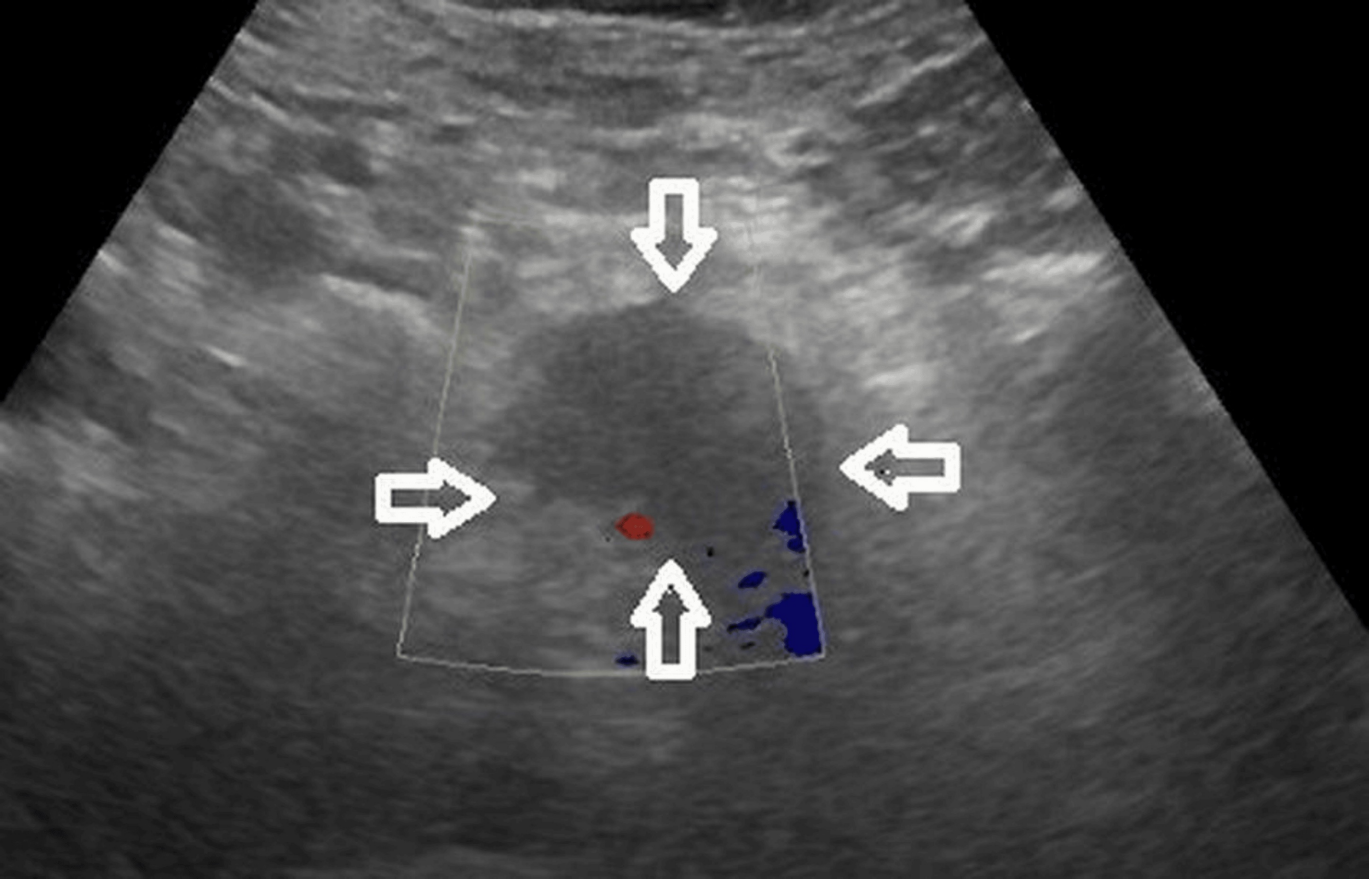
Abdominal ultrasound (US) depicting a hypoechoic pancreatic mass in the head and body of the pancreas (arrows), measuring 5.8 × 3.7 × 3.9 cm

A 52-year-old Hispanic male with a history of hypertension, type 2 diabetes mellitus, hyperlipidemia, coronary artery disease with stent placements, and obstructive sleep apnea presented to the Emergency Department (ED) with left upper quadrant abdominal pain lasting one week. The pain was rated as 7 out of 10, radiating to the epigastric region and mid-back, and accompanied by abdominal bloating and nausea. He denied vomiting, weight loss, severe weakness, diarrhea, or fever. On physical examination, vital signs were normal. The patient had no jaundice. The left upper abdomen was mildly tender without a palpable mass, and the rest of the physical exam was unremarkable. Laboratory test results showed a normal complete blood count, a normal metabolic panel, slightly elevated alkaline phosphatase (147 units/L), normal alanine transaminase and aspartate aminotransferase, and a normal bilirubin level. Abdominal ultrasound depicted a hypoechoic pancreatic mass in the head and body of the pancreas, measuring 5.8 × 3.7 × 3.9 cm (Figure 1).

**Figure 2:**
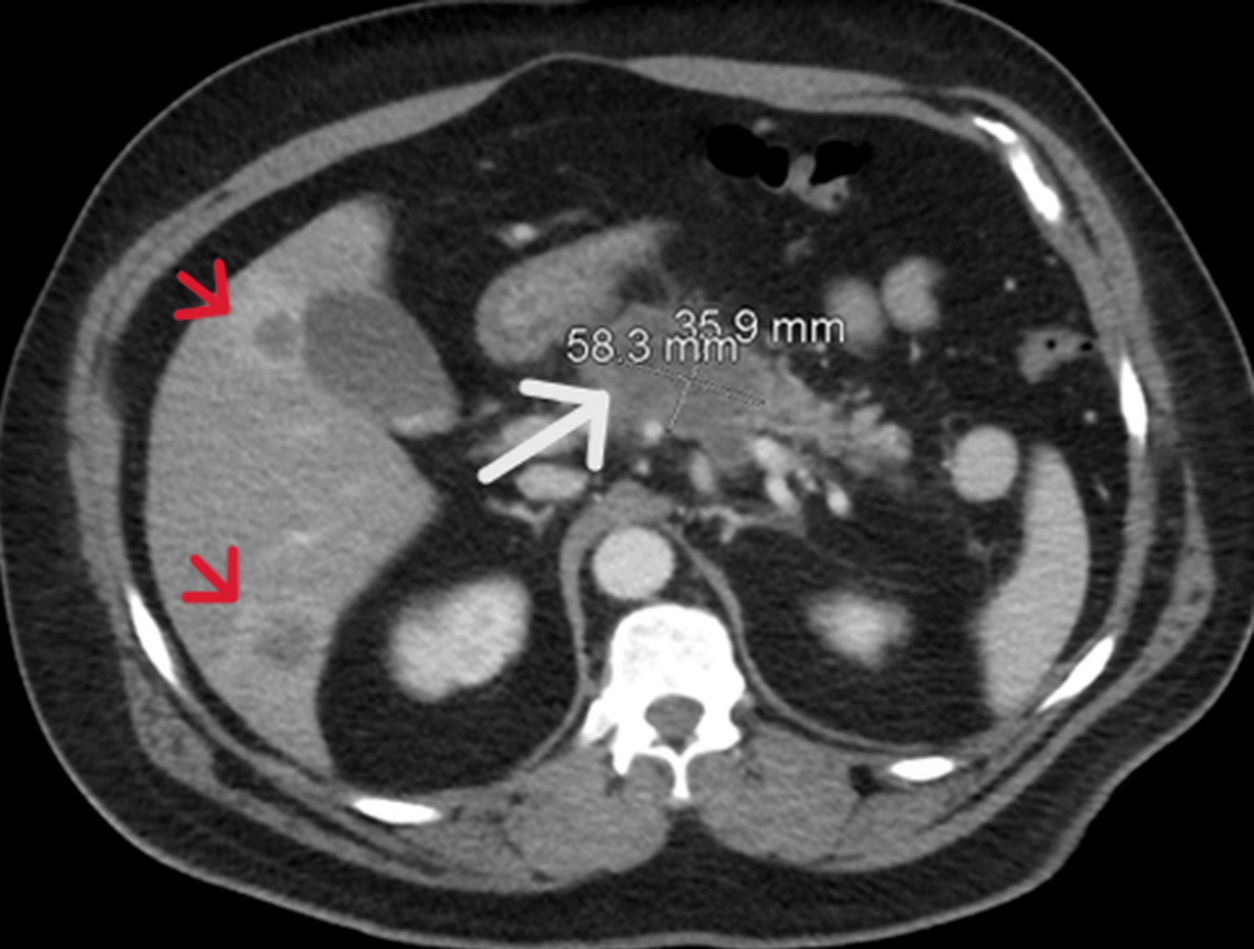
Computed tomography (CT) scan of the abdomen with contrast, revealing a hypodense pancreatic mass (white arrow) and multiple enhancing low-density liver lesions (red arrows)

A computed tomography (CT) scan of the abdomen and pelvis with contrast revealed a hypodense pancreatic mass, with multiple enhancing low-density liver lesions (Figure 2).

**Figure 3:**
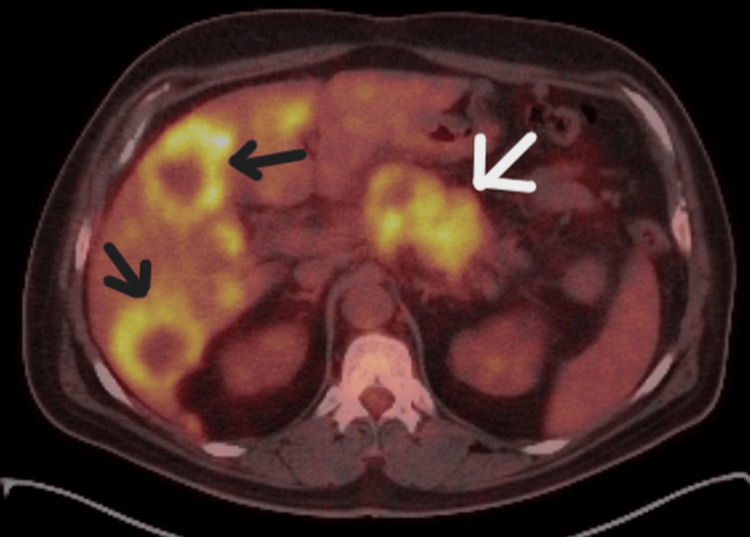
Positron emission tomography/computed tomography (PET/CT) scan showing a hypermetabolic pancreatic tumor (white arrow) and metastatic liver lesions (black arrows)

Positron emission tomography (PET)/CT showed a mass in the head and body of the pancreas, and hypermetabolic lesions in both liver lobes (Figure 3) and periaortic lymph nodes.

An ultrasound-guided needle biopsy of the hepatic lesion confirmed PDAC with focal squamous differentiation. After discussing options with the patient, chemotherapy was started. Due to his extensive cardiac history, the patient was initiated on FOLFOX every two weeks. CA 19-9 levels were monitored throughout treatment to assess response (Table 1).

**Table 1:** CA 19-9 response to FOLFOX chemotherapy

Day	CA 19-9 (Normal: <34 U/L)	FOLFOX Chemotherapy
1	2,344	-
21	4,057	-
63	-	Cycle 1
70	25,452	-
74	-	Cycle 2
80	31,084	-
88	-	Cycle 3
90	58,560	-
94	51,393	-
102	-	Cycle 4
108	91,083	-
115	98,646	-
116	-	Cycle 5
122	96,233	-
135	-	Cycle 6
138	59,263	-
143	60,309	-
149	-	Cycle 7
157	61,818	-
163	-	Cycle 8
171	66,172	-
177	-	Cycle 9
185	62,673	-

**Figure 4:**
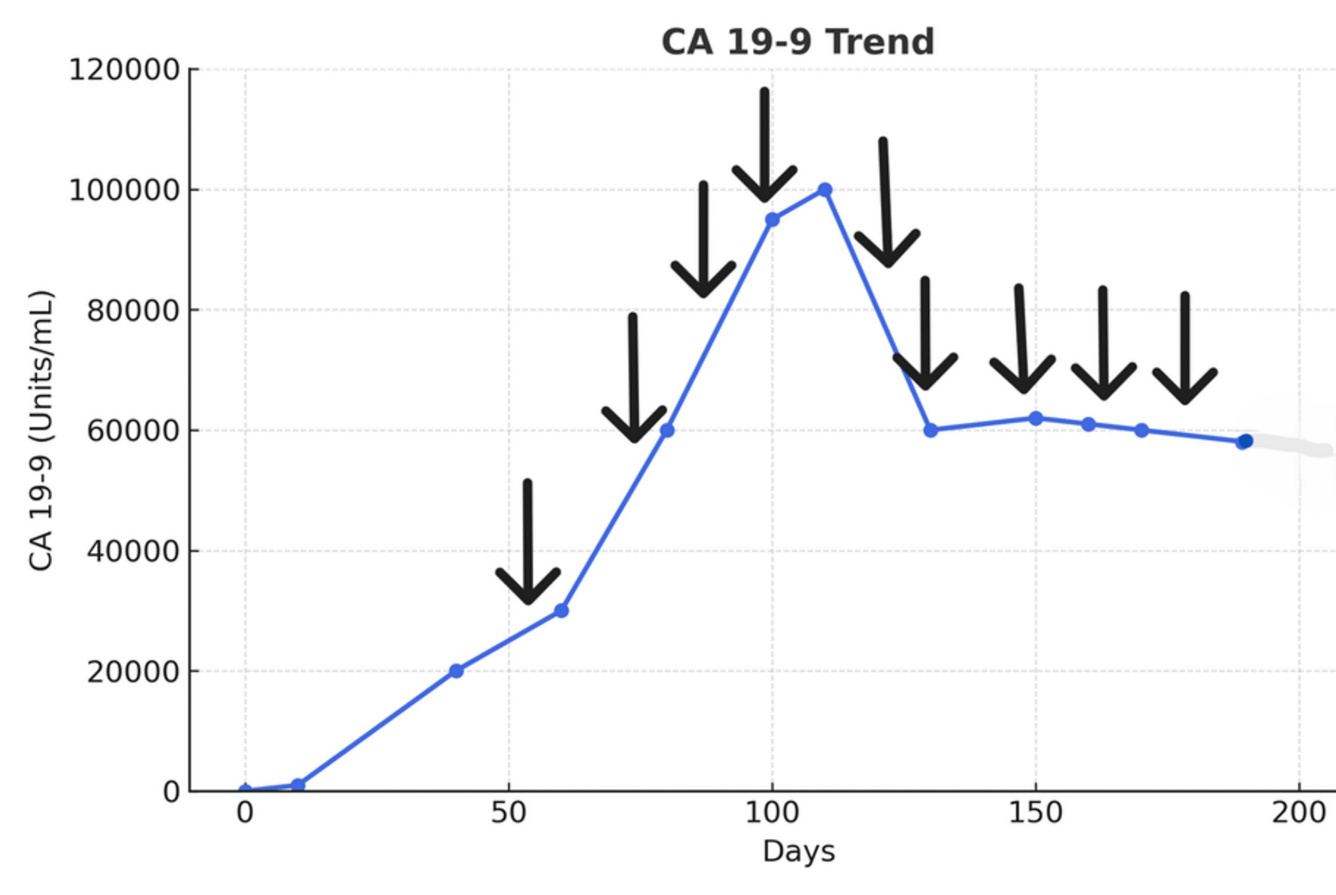
Biochemical pseudoprogression: the CA 19-9 levels continued rising even after four cycles of chemotherapy

The initial CA 19-9 measurement (Day 1) was 2,344 units/mL (normal <37 units/mL), and levels gradually increased to 25,452 units/mL by the first chemotherapy cycle (Day 70). Despite chemotherapy, CA 19-9 levels continued to rise (Figure 2). After four cycles (two months into treatment), CA 19-9 levels exceeded 90,000 units/mL. Without other conditions that could elevate CA 19-9, such as gallstones, cholangitis, or hepatitis, and with bilirubin levels within normal limits, a decision had to be made whether to change chemotherapy, as rising CA 19-9 levels usually indicate disease progression. However, the patient reported no abdominal pain and felt generally better, despite the increased CA 19-9 levels. He had more energy and regained appetite and body weight. Pseudoprogression was therefore suspected, and the same FOLFOX chemotherapy was continued. After the sixth cycle (three months after initiation), CA 19-9 levels began to decrease. After the seventh cycle, CA 19-9 dropped to 59,265 units/mL and plateaued around 60,000 units/mL (Figure 4). The patient was able to resume activities such as fishing and shared his catch with other cancer patients.

## Discussion

Pseudoprogression occurs when imaging or biomarker assessments suggest tumor progression, despite stable or improving cancer status [4]. It was first described in glioblastoma patients treated with radiation and temozolomide chemotherapy [7], but is more commonly associated with ICI immunotherapies that can induce inflammatory responses mimicking disease progression [4].

Pseudoprogression has been described in various tumors, primarily melanoma, as well as non-small cell lung cancer, renal cancer, urothelial cancer, and head and neck squamous cell cancer [4-7], with an incidence of about 8.8%, depending on cancer type. However, pseudoprogression has not been documented in pancreatic cancer cases [4-6,8,9].

The molecular mechanism is not fully understood, but immune cell infiltration, cytokine release, and subsequent inflammation may contribute to temporary tumor swelling [4,9]. In experimental pancreatic cancer models, stimulation of tumor-associated stroma has been suggested as a mechanism, making the tumor area appear more prominent [10].

Biochemical pseudoprogression may differ, potentially caused by cancer cell destruction from chemotherapy, releasing tumor markers into the bloodstream. If the tumor marker has a long half-life, levels may remain high, giving the impression of progression, even if the cancer is responding to treatment. However, the half-life of CA 19-9 is relatively short, around one day [11].

Pseudoprogression presents challenges in cancer treatment, complicating assessment of treatment response and potentially leading to premature treatment changes in patients who are actually improving. Conventional imaging may not always distinguish inflammatory changes from tumor growth, though functional magnetic resonance imaging (MRI) and PET may help identify pseudoprogression [12,13]. For instance, 18F-fluciclovine PET has been useful in detecting pseudoprogression in glioblastoma [14], whereas conventional 18F-FDG PET/CT has limitations [15].

Most patients with pseudoprogression have a good performance status and are paucisymptomatic [4]. In our patient, abdominal pain subsided, and physical performance improved, supporting pseudoprogression as a consideration.

## Conclusions

This case highlights a rare example of biochemical pseudoprogression in PDAC, where CA 19-9 levels initially increased during FOLFOX chemotherapy, but subsequently declined with ongoing treatment. Clinical improvement, despite rising tumor markers, underscores the importance of interpreting CA 19-9 trends within the broader clinical context to avoid prematurely misclassifying treatment response as true disease progression.
